# Interplay Between the Object and Its Symbol: The Size-Congruency Effect

**DOI:** 10.5709/acp-0191-7

**Published:** 2016-06-30

**Authors:** Manqiong Shen, Jiushu Xie, Wenjuan Liu, Wenjie Lin, Zhuoming Chen, Fernando Marmolejo-Ramos, Ruiming Wang

**Affiliations:** 1 Center for Studies of Psychological Application, Guangdong Provincial Key Laboratory of Mental Health and Cognitive Science, School of Psychology, South China Normal University, Guangzhou, China; 2 Language Disorder Center, The First Affiliated Hospital of Jinan University, Guangzhou, China; 3 Gösta Ekman Laboratory, Department of Psychology, Stockholm University, Stockholm, Sweden

**Keywords:** grounded cognition, perceptual symbol theory, conceptual processing, perceptual processing, size congruent effect

## Abstract

Grounded cognition suggests that conceptual processing shares cognitive resources with perceptual processing. Hence, conceptual processing should be affected by perceptual processing, and vice versa. The current study explored the relationship between conceptual and perceptual processing of size. Within a pair of words, we manipulated the font size of each word, which was either congruent or incongruent with the actual size of the referred object. In Experiment 1a, participants compared object sizes that were referred to by word pairs. Higher accuracy was observed in the congruent condition (e.g., word pairs referring to larger objects in larger font sizes) than in the incongruent condition. This is known as the size-congruency effect. In Experiments 1b and 2, participants compared the font sizes of these word pairs. The size-congruency effect was not observed. In Experiments 3a and 3b, participants compared object and font sizes of word pairs depending on a task cue. Results showed that perceptual processing affected conceptual processing, and vice versa. This suggested that the association between conceptual and perceptual processes may be bidirectional but further modulated by semantic processing. Specifically, conceptual processing might only affect perceptual processing when semantic information is activated. The current study

## Introduction

We know that a radish is larger than a sesame seed. However, if we displayed the words *radish* and *sesame seed* in different font sizes, would that affect our understanding of these words? For instance, would we process the word *radish* faster when the word is presented with a larger rather than a smaller font size? Similarly, would we process the word *sesame seed* faster when the word is presented with a smaller font size than a larger one? In the present study, we tested whether the relationship between perceptual and conceptual processing of size is symmetric. Then, we explored factors affecting the bidirectional connection between conceptual and perceptual processing of size.

 Conceptual processing plays an essential role in cognitive processing (Murphy, 2002). People may describe their thoughts as mental images, sizes, weight sensations, imagined movements through space, simulated sequences of actions, and so on (Kaspar & Vennekötter, 2015). The idea that the elements of thought consist of visual and motor images is at the core of grounded cognition theory (e.g., Barsalou, 1999; Glenberg, 1997; Grush, 2004; Zwaan, 1999). Among theories of conceptual representation, one representative view of grounded cognition is the perceptual symbol theory (PST), which proposes that conceptual representation relies on the sensory-motor system (Barsalou, 1999). Foundations of mental representation are perceptual symbols, which are partial reinstatements of the neural patterns that are stored in perceptual and motor brain areas during actual experience and interaction with the environment (Pecher, Boot, & Van Dantzig, 2011). PST, which describes bidirectionally grounded effects, claims that conceptual representation and perceptual systems share the same resources (Barsalou, 1999; Slepian & Ambady, 2014). Hence, conceptual processing is affected by perceptual processing, and vice versa. 

 Ample studies have supported the notion that perceptual processing affects conceptual processing. Zwaan and Yaxley (2003a, 2003b) examined whether the locations of word pairs affected semantic judgments. One word of each pair referred to objects at higher locations while the other referred to objects at lower locations (e.g., *attic*, *basement*). These word pairs were presented in an congruent relation (e.g., *attic* presented above *basement*) or in an incongruent relation (e.g., *basement* above *attic*). Participants judged whether these words were related. The results showed that when word pairs were presented in an incongruent relation, participants’ responses were significantly slower than in the congruent relation condition (but see Louwerse, 2008). Similar results were found by Van Dantzig, Pecher, Zeelenberg, and Barsalou (2008) , who asked participants to finish a perceptual detection task before a property veriﬁcation task. In the perceptual detection task, a stimulus referring to the auditory, visual, or tactile modality was presented to the left or right side of the display or participants. Participants responded to the presentation location of the stimulus. In the property verification task, participants read short sentences and judged sentence logic when concepts were modified by adjectives. These sentences also referred to the same or to a different sensory modality as the perceptual detection task (e.g., “a bee buzzes”, “a banana is yellow”, “a coin is hard”). Participants’ responses in the property veriﬁcation task were slower for those trials that were preceded by perceptual trials in a different modality than those that were preceded by perceptual trials in the same modality. This switching effect between perceptual and conceptual processing supported the hypothesis that perceptual and conceptual representations were partially based on the same system. 

 Meanwhile, some studies have revealed that conceptual processing affects perceptual processing. Richter and Zwaan (2009) used a semantic priming paradigm to investigate whether color representations were activated when color words were processed. Participants first saw a color square; then, a color word, a non-color word, or a non-word was shown in black letters within a white rectangle in the center of the screen, followed by another color square. Participants judged whether the word was meaningful (i.e., a lexical decision task) and whether the second color square was the same as the first. The color words were either matched or mismatched with the color squares. Results showed that participants’ responses were faster in the lexical decision task when color words were congruent with color squares. These findings are consistent with the experiential view of language comprehension according to which color perception and the comprehension of color words are based on overlapping representational resources. 

 In sum, many studies have found that conceptual processing is affected by perceptual processing (Pecher, Zeelenberg, & Barsalou, 2003; Van Dantzig et al., 2008; Zwaan & Yaxley, 2003a, 2003b) and vice versa (Graf, Uttl, & Tuokko, 1995; Hentschel, 1973; MacLeod, 1991; Palef & Olson, 1975; Richter & Zwaan, 2009; Windes, 1968). Although perceptual processing has a role in the processing of concepts, semantic activation significantly affects conceptual processing. Semantic activation implicates the re-enactment of sensorimotor information which, in turn, impinges upon perceptual processing. When a word is processed in a conceptual task, its formations are first activated and then a situated simulation related to its meaning including information about perception, action, and mental states is activated. Moreover, the word sets off a situated simulation to represent its meaning only when the level of semantic activation is sufficient (Barsalou, 1999; Glenberg & Kaschak, 2002). 

 Previous studies have supported that semantic activation significantly affects conceptual processing. For example, D’Arcais, Schreuder, and Glazenborg ( 1985) tested the activation of semantic information, including perceptual and functional information. Semantic information during word recognition includes two components; a perceptual and a non-perceptual component. These authors found that there was a different rate of activation in the semantic information of a word. For example, processing the word pair *cherry-apple* was responded to faster than *cherry-banana* because *cherry* and *apple* were similar both in perception (both have a round appearance) and function while *cherry* and *banana* were only similar in function. Perceptual semantic information probably is acquired earlier in the process of the acquisition of words’ meanings. Thus, it seems to be available earlier or with faster maximum activation than information based on abstract or functional properties of the objects to which the words refer. Lindemann, Stenneken, van Schie, and Bekkering (2006) used a go/no-go paradigm to investigate the activation of semantic information during action preparation. Participants were asked to grasp an object (e.g., a cup) or lift a finger in association with the object’s position. Word stimuli were consistent to the action goals of the object use or to the finger lifting. Results showed that a double dissociation of consistency effects was present for semantic categorizations, but it disappeared when a letter identification task was introduced. The findings indicated that semantic knowledge was activated during action preparation. In sum, semantic activation, according to the task, played a significant role in conceptual processing. Thus, in our study, we adopted different tasks to test the bidirectional relationships between conceptual and perceptual processing and then to test the role of semantic activation in these relationships. 

 Previous studies have investigated the concept of size (Gabay, Leibovich, Henik, & Gronau, 2013; Paivio, 1975; Rubinsten & Henik, 2002). However, this symmetric concern on the processing of size is under debate (Gabay et al., 2013; Paivio, 1975; Rubinsten & Henik, 2002). Paivio, for example, presented participants with pairs of pictures or words referring to larger or smaller animals. Pictures and words were presented in large or small sizes. Participants chose the pictures or words that referred to larger animals in each pair. Results showed that participants responded faster when the animals’ real sizes were congruent with the presentation sizes (i.e., large animals were presented in large pictures; small animals, in small pictures) than when they were incongruent. This result is known as the size-congruency effect. However, the same effect was not found when participants judged words. On the contrary, Rubinsten and Henik found a size-congruency effect with words. They presented word pairs that referred to larger or smaller animals in large or small font sizes. Participants judged which words referred to larger animals (i.e., semantic judgment) or which font sizes were larger. Results revealed the size-congruency effect for both semantic and font size judgments. 

 Based on the opposite findings of Paivio (1975) and Rubinsten and Henik (2002) , we assumed that semantic processing might modulate the influence. Louwerse and Jeuniaux (2010) explored the linguistic and embodied nature of conceptual processing. Participants made quick judgments about whether pairs of words or pictures were semantically related or had a congruent relationship (e.g., *attic* presented *above basement*). It was found that embodiment improved participants’ performance in congruency judgments for pictures while linguistic processing improved participants’ performance in semantic judgments for words. For example, Hoedemaker and Gordon (2014) used a priming paradigm to explore whether the activation of magnitude information about semantic size associated with lexical items was encoding-based or goal-based. Triplets of numbers, object, and animal names were presented. The results showed that the activation of numerical magnitude representations was encoding-based as well as goal-driven, while the activation of a word’s size information was goal-driven and did not occur automatically during encoding. Hence, task-related factors might have an effect on the relationship between conceptual and perceptual processing (Huber, 1985; Kaspar & Vennekötter, 2015; Louwerse & Connell, 2011; Robinson, 2001). 

The present study aimed to test the directional association between conceptual and perceptual processing. Specifically, we manipulated the font size of words that referred to large or small objects in reality to create congruent (e.g., words that referred to large objects were presented in relatively large fonts) or incongruent (e.g., words that referred to large objects were presented in relatively small fonts) conditions. According to PST, conceptual processing relies on the activation of the sensory-motor system. Similar neural firing patterns should occur whether a person is processing a pair of words, like *basketball* and *coin*, or the person is seeing those objects in reality. Specifically, the visual system signals seeing a basketball and a coin; the motor system signals the actions of grasping, playing, and picking up. This process may affect how people judge words which indicate objects of various sizes in reality. Hence, conceptual processing would affect perceptual processing, and vice versa.

 To test the relationship of conceptual and perceptual processing, we employed a Stroop-like paradigm. Experiment 1a required participants to judge which of two objects indicated by two words was larger/smaller in reality (i.e., referred-object size judgment task). We predicted a size-congruency effect which would suggest that conceptual processing was influenced by perceptual processing (Paivio, 1975). In Experiment 1b, the procedure was the same, except that a font judgement task replaced the referred-object size judgment task. Since we assumed that semantic activation might affect the size-congruency effect and semantic processing in Experimen*t*_1_b would not be sufficiently strong, we did not predict the size-congruency effect in this experiment. To examine our explanation for the absence of the size-congruency effect in Experiment 1b, we added a recognition task to increase semantic activation in Experiment 2. We predicted a size-congruency effect in this experiment which would support our semantic hypothesis. The results might also support our assumption that perceptual processing would be affected by conceptual processing. Experiment 3 further enhanced semantic activation; a mixed task in Experiment 3a and a dual task in Experiment 3b required participants to perform a semantic or visual judgment task, randomly changing from trial to trial. We predicted a size-congruency eﬀect in both these tasks or only in the semantic judgment task. All in all, the level of semantic activation might play an important role in size-congruency effect (Rubinsten & Henik, 2002). 

## Experiment 1

### Method

#### Participants

In Experiment 1a, forty undergraduate and postgraduate students (*M*_age_ = 22 years, *SD* = 2.2, 32 were female) from South China Normal University, Guangzhou, China, participated in this experiment. Another 40 students (*M*_age_ = 20.20 years, *SD* = 1.43, 32 were female) from the same university participated in Experiment 1b. All participants had normal or corrected-to-normal vision. They were paid after the experiment. All the experiments reported here were approved by the ethics review board of South China Normal University.

#### Materials

 For both Experiments 1a and 1b, participants sat at a distance of 57 cm from the display (31 × 24 cm). E-prime 1.2 was used for presenting stimuli and recording participants’ responses (Schneider, Eschman, & Zuccolotto, 2002). 

 Forty-eight Chinese words were used for the stimuli in Experiment 1a (see Appendix). Stimuli consisted of 24 pairs of object names which were chosen from the *Modern Chinese Dictionary* (Wang, Fang, & Zuo, 1995). Both items in each pair came from the same category (e.g., fruits, artifacts, etc.). In each word pair, one word referred to the object (hereafter known as “referred object”) that was larger in reality than the other referred object (e.g., *sesame seed-radish* or *grape-watermelon*). In a pretest, five independent raters correctly classified the larger/smaller item of each pair in 100% of all cases (Connell, Lynott, & Dreyer, 2012). All materials consisted of two Chinese characters, and there was no significant difference in stroke numbers between words that referred to larger and smaller objects, *t*(23) = 0.19, *p* = 0.852. Another 20 students (*M*_age_ = 20.55 years, *SD* = 1.82 years, 11 were female) from South China Normal University, Guangzhou, China, rated the familiarity of these word pairs on a 7-point scale (1 = unfamiliar/unknown, 7 = familiar/very well known). All rating scores were higher than 5 (*M* = 6.43; *SD* = 0.31) ( Setti, Caramelli, & Borghi, 2009; Wang & Zhang, 2014). 

In the experiment, the words referring to big and small objects were presented in large and small font size, which was counterbalanced between participants. All objects appeared in both congruent/incongruent trials. The assignment of the correct answer to the left/right side of the screen and the order of the two tasks (i.e., judging the larger or the smaller object first) were counterbalanced between participants. Overall, there were eight combinations (2 congruent/incongruent × 2 left/right × 2 small/large task) in Experiment 1a. All items were repeated once for each participant. The experiment consisted of 64 trials in total: 16 practice trials and 24 trials for each experimental condition of congruency (congruent/incongruent).

#### Procedure

In Experiment 1a, the experiment was divided into two blocks that were counterbalanced between participants. Half of the participants first judged which referred object size was larger than the other, while the remaining half first judged which referred object size was smaller than the other.

**Figure 1.**
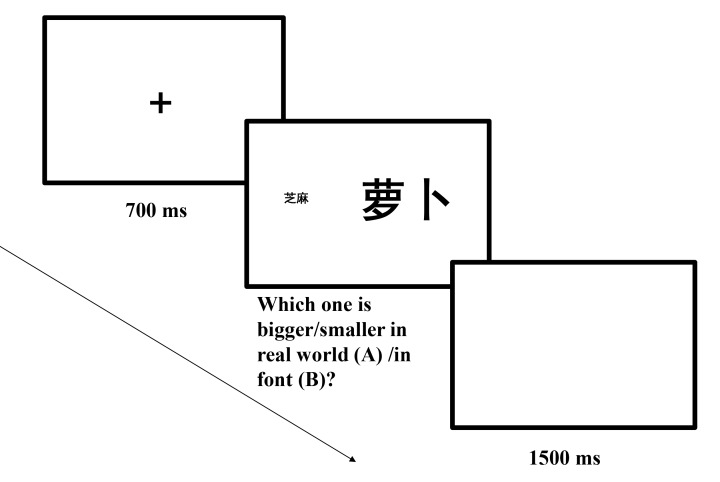
Sequence of events in a trial of Experiment 1a (A) and Experiment 1b (B) (萝 卜 means *radish* and 芝 麻 means *sesame seed*).

All materials were presented on a white background. Each trial began with a red fixation cross at the center of the screen for 700 ms. After that, a pair of Chinese words (e.g., *sesame seed*, *radish*; in boldface font) appeared horizontally on the screen. One word was presented in a larger font size (144-points), while the other word was presented in a smaller font size (36-points). Each word pair appeared at the center of the left and right halves of the screen. These word pairs were presented for 5 s or until the participants responded. Participants were asked to put their left index finger on the *c* key and their right index finger on the *m* key. The stimulus-response mapping rule was counterbalanced. Participants were asked to respond as quickly and accurately as possible. After 1,500 ms, the next trial started. Eight practice trials were conducted prior to the experiment (see Figure 1A).

The procedure for Experiment 1b was the same as for Experiment 1a, except that participants judged which item was presented in a larger/smaller font size. Participants were asked to read the words, then judged which word had the larger/smaller font size (see Figure 1B).

#### Design and statistical analyses

 Experiments 1a and 1b used a single factor within-subjects design (congruency between referred object sizes and font sizes: congruent vs. incongruent). The dependent variables were the participants’ reaction times (RTs) and accuracy rates (ARs). Data from two participants were removed because of low accuracy (< 80%). Only RTs for correct trials were analysed; outliers were dealt with by removing RTs above two SD (see Marmolejo-Ramos, Cousineau, Benites, & Maehara, 2015). In the case of AR, all data were analysed. Data from participants with lower ARs were removed from the analyses. To analyse the data in a by-subject and by-item fashion, *t*-tests were used. Cohen’s d effect sizes are reported for the pairwise comparisons (see Lakens, 2013). Beanplots were used to graphically report the results (Kampstra, 2008). 

### Results

In Experiment 1a, data from two participants were removed because of low accuracy (< 80%). For the remaining participants, RT data from the task with erroneous trials (32 trials, 3.50%) were discarded. RTs beyond two SD were also excluded from further analyses (5.70%). In Experiment 1b, RT data with erroneous trials were deleted (0.46%), and RTs beyond two SD were excluded from the analyses (3.90%). All AR data were included for further analyses.

**Figure 2.**
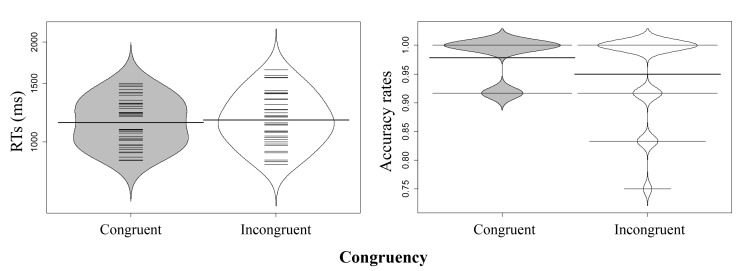
Distribution of participants’ reaction times (RTs) (ms) and accuracy rates (ARs) in Experiment 1a (the thick horizontal lines represent the mean; the thin horizontal lines correspond to individual observations; and the grey and white areas display the data’s distribution). RT: Congruent; *M* = 1157.27, *SE* = 30.67. Incongruent; *M* = 1183.71, *SE* = 35.25. AR: Congruent; *M* = 0.978, *SE* = 0.006. Incongruent; *M* = 0.949, *SE* = 0.012.

**Figure 3.**
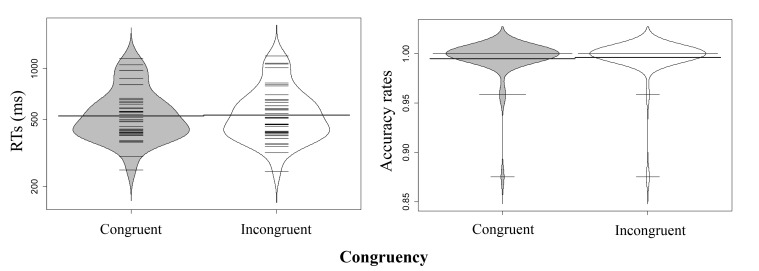
Distribution of participants’ reaction times (RTs) (ms) and accuracy rates (ARs) in Experiment 1b (conventions as in Figure 2). RT: Congruent; *M* = 558.05, *SE* = 33.41. Incongruent; *M* = 565.45, *SE* = 35.07. AR: Congruent; *M* = 0.994, *SE* = 0.003. Incongruent; *M* = 0.995, *SE* = 0.003.

Trimmed RT and AR data were submitted to a paired *t*-test taking both participants (*t*_1_) and items (*t*_2_) as random factors. In Experiment 1a, there was no significant main effect of congruency in the RT analysis, *t*_1_(1, 37) = 1.37, *p* = .178, *d* = 0.13; *t*_2_(1, 23) = 1.55, *p* = .136, *d* = 0.36. However, in the AR analyses, the main effect of congruency was significant, *t*_1_(1, 37) = 2.48, *p* = .017, *d* = 0.48; *t*_2_(1, 23) = 2.21, *p* = .037, *d* = 0.56. ARs were higher in the congruent than in the incongruent condition (see Table 1, Figure 2). Hence, we found there was a size-congruency effect and that conceptual processing was influenced by perceptual processing. In Experiment 1b, the main effect of congruency was neither significant in the RTs, *t*_1_(39) = 1.424, *p* = .162, *d* = 0.034; *t*_2_(23) = 0.038, *p* = .970, *d* = 0.015, nor AR analyses, *t*_1_(39) = 0.216, *p* = .830, *d* = 0.048; *t*_2_(23) = 0.296, *p* = .770, *d* = 0.090^1^ (see Table 2, Figure 3).

In addition, we conducted an analysis of variance (ANOVA) to test the interaction of task and congruency. Task (referred-object judgments in Experiment 1a vs. font size judgments in Experiment 1b) was included as a between-subjects factor in the analyses. In RT analyses, the interaction between task and congruency was not significant, *F*_1_(1, 76) = 0.951, *p* = .332, η_p_^2^ = .012; *F*_2_(1, 46) = 0.905, *p* = .346, η_p_^2^ = .019; while in AR analyses, it was significant, *F*_1_(1, 76) = 6.160, *p* = .015, η_p_^2^ = .075; *F*_2_(1, 46) = 5.054, *p* = .029, η_p_^2^ = .099. Planned, simple effect analyses found that participants’ accuracy was significantly higher in the congruent than in the incongruent condition in the referred-object judgments task, *F*_1_(1, 76) = 11.23, *p* = .001, η_p_^2^ = .153; *F*_2_(1, 46) = 9.29, *p* = .004, η_p_^2^ = .166, but not in the font size judgments task, *F*_1_(1, 76) = 0.010, *p* = .907, η_p_^2^ < .001; *F*_2_(1, 46) = 0.020, *p* = .897, η_p_^2^ < .001. This indicates that the size-congruency effect was modulated by tasks.

### Discussion

The AR data of this experiment demonstrated a size-congruency effect. When the font sizes and referred object sizes were congruent, participants’ responses were more accurate than when they were incongruent. This indicated that font size affected processing of size information of concepts.

 In Experiment 1a, participants performed a semantic task (i.e., judged the larger/smaller referred-to objects). Font sizes of target words had nothing to do with the task. Hence, the influence of perceptual processes on the conceptual judgment likely occurred automatically. These results were congruent with the results of Rubinsten and Henik (2002) . They found faster semantic judgments for congruent than incongruent stimuli. Hence, we conceptually replicated these findings successfully and found a size-congruency effect. 

In the AR results of Experiment 1a’s semantic task, perceptual processing affected conceptual processing. However, Experiment 1b’s font size task showed no size-congruency effect. In terms of difficulty, the font judgment task of Experiment 1b was easier than the semantic task, and participants were more likely to ignore the word meaning, indicating that semantic activation might moderate the size-congruency effect. Therefore, in Experiment 2, we added a task requiring participants to process the meaning of the presented words. This forced them to process semantic information.

## Experiment 2

### Method

#### Participants

Another 32 university students (*M*_age_ = 21 years, *SD* = 1.54, 16 were female) from South China Normal University, Guangzhou, China, participated in this experiment. All other aspects were same as in Experiment 1a.

#### Materials and procedure

**Figure 4.**
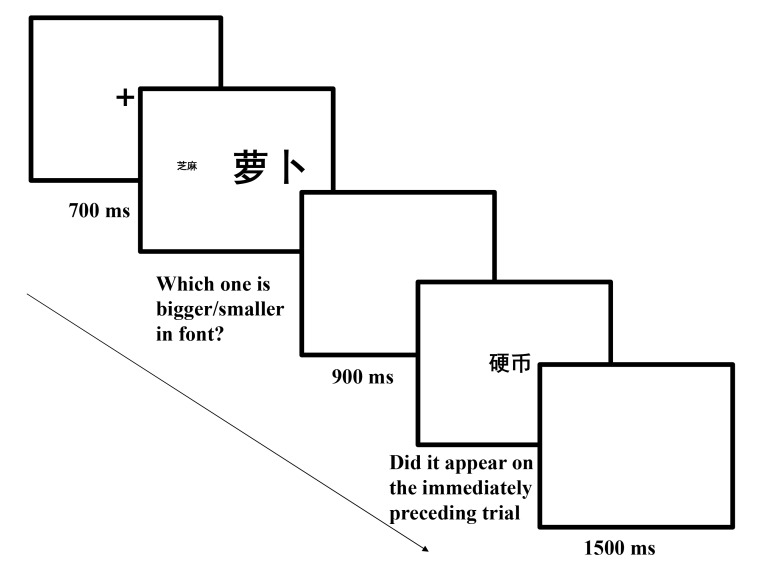
Sequence of events in a trial of Experiment 2 (萝 卜 means *radish* and 芝 麻 means *sesame seed*).

Materials and procedure were the same as in Experiment 1b, with these exceptions: Participants were required to perform a font judgment task and a word recognition task sequentially. Given that the font judgment task may be too easy, we added a word recognition task to make participants pay attention to word meanings. Participants first judged which word had larger/smaller font size. After they responded, a blank screen was presented for 900 ms. Then, a word that either had or had not appeared on the immediately preceding trial was presented at the center of the screen. In half of the trials, the words were the same as those in the size judgment task. In the remaining half, the words belonged to the same category as the word pairs in the size judgment task but had not appeared previously. Participants judged whether this word had been presented before; they pressed *c* or *m* keys for “yes” or “no” responses, respectively (see Figure 4).

#### Design and statistical analyses

We employed a single factor within-subject design (congruency between referred object sizes and word font sizes: congruent vs. incongruent). The statistical analyses were the same as those used for Experiment 1a.

### Results

Data from five participants were removed because of low accuracy (< 80%) in the word recognition task. To improve power and validity, we collected another five participants’ data in the second round. Finally, there were 32 valid participants’ data in the final analyses. RT data with erroneous trials were deleted (5.18%), and RTs beyond two SD were excluded from the analyses (4.05%). All AR data were included for further analysis.

**Figure 5.**
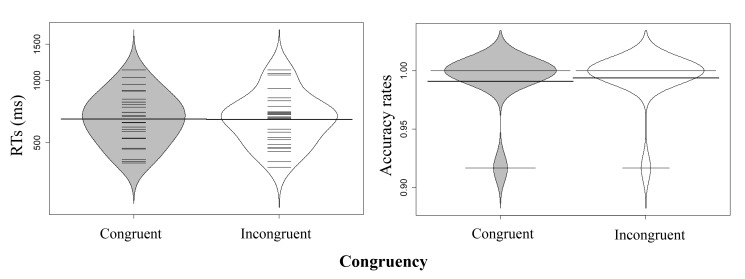
Distribution of participants’ reaction times (RTs) (ms) and accuracy rates (ARs) in Experiment 2 (conventions as in Figure 2). RT: Congruent; *M* = 677.61, *SE* = 33.04. Incongruent; *M* = 680.70, *SE* = 34.23. AR: Congruent; *M* = 0.992, *SE* = 0.004. Incongruent; *M* = 0.994, *SE* = 0.003.).

Trimmed RT and AR data of the font judgment task were submitted to a paired *t*-test taking both participants (*t*_1_) and items (*t*_2_) as random factors. The main effect of congruency was neither significant in the RTs, *t*_1_(31) = 0.25, *p* = .799, *d* = 0.094; *t*_2_(23) = 0.36, *p* = .719, *d* = 0.04, nor in the AR analyses, *t*_1_(31) = 0.44, *p* = .662, *d* = 0.11; *t*_2_(23) = 0.36, *p* = .719, *d* = 0.13 (see Table 3, Figure 5).

### Discussion

To ensure that participants paid attention to word meaning, we included a recognition task in Experiment 2. However, the size-congruency effect was still absent. Given that RTs in the current experiment were nearly twice as fast as RTs in Experiment 1a; participants might just process the word form and ignore the words’ semantic meanings. This might be one reason for the absence of the size-congruency effect. Hence, in the following experiments, in order to improve the semantic activation, we simultaneously adopted the referred-object size judgment task used in Experiment 1a and the font size judgment task used in Experiment 1b. These two tasks were mixed in the following experiment to make sure that participants paid enough attention to the word meanings. We were interested in whether conceptual processing interacts with perceptual processing and whether the relationship is bidirectional under certain conditions.

## Experiment 3a

### Method

#### Participants

Thirty-two university students (*M*_age_ = 20.75 years, *SD* = 1.626, 24 were female) from South China Normal University, Guangzhou, China, participated in this experiment. Other aspects were the same as in Experiment 2.

#### Materials and Procedure

Materials were identical to those of Experiment 1. The words referring to big and small objects were presented in large or small font size, which was counterbalanced between participants. At the same time, all objects were presented in the congruent or incongruent trials, and the assignment of the correct answer to the left/right side of the screen was counterbalanced between participants. Tasks changed randomly from trial to trial. Thus, in Experiment 3a, there were 16 combinations (2 congruent/incongruent × 2 left/right × 2 small task/large task × 2 referred-object judgment task/font size judgment task). The experiment consisted of 64 trials in total: 16 trials for practice and 12 trials for each combination condition.

The experiment was divided into two blocks that were counterbalanced between participants. Half of the participants first judged which word was represented in the larger referred object/font size, while the remaining half first judged which word was presented in the smaller size.

**Figure 6.**
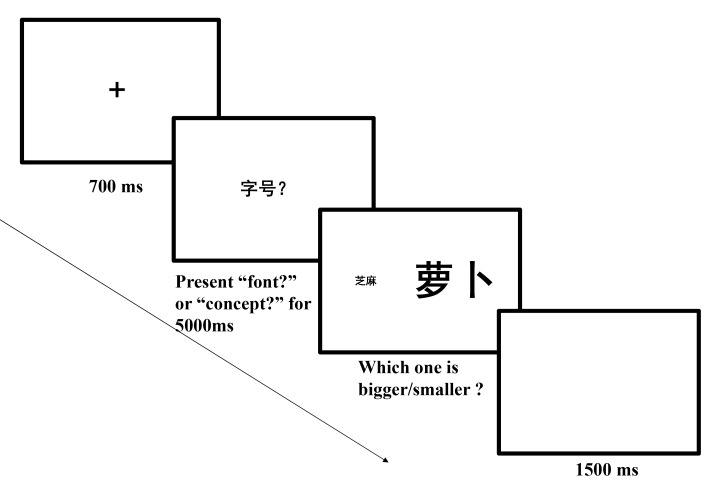
Sequence of events in a trial of Experiment 3a (萝 卜 means *radish*, 芝 麻 means *sesame seed* and 字 号 means font?).

Each trial began with a red fixation cross that was presented for 700 ms at the center of the screen. Then, a task cue (i.e., “concept?” or “font?”) appeared at the center of the screen for 1,500 ms, which informed participants to either judge the referred object’s size or the font size. After that, a word pair appeared at the center and then participants responded. If participants had seen “concept?”, they judged which word referred to the larger or smaller object in reality. If participant had seen “font?”, they judged which word was presented in larger or smaller font size. Half of the participants first judged which object was smaller, and the other half first judged which one was bigger. The word pairs were presented for 5 s or until participants responded. Participants pressed the *c* or m key on the keyboard to judge which word referred to a larger/smaller object in reality or which word was presented in a larger/smaller font size. The allocation of the response keys to larger or smaller labels was counterbalanced between participants. After a 1,500 ms blank screen, the next trial was presented (see Figure 6).

#### Design and statistical analyses

The study employed a 2 (task: referred-object judgment task vs. font size judgment task) × 2 (consistency: congruent vs. incongruent) within-subject design. The statistical analyses were the same as those used for Experiment 1a.

### Results

RT data with erroneous trials were deleted (3.71%), and RTs beyond two SD were excluded from the analyses (5.27%). All AR data were included in further analyses.

Trimmed RTs and ARs were submitted to two separate, two-way repeated measures ANOVAs with task (referred-object size judgment vs. font size judgment) and congruency (congruent vs. incongruent) as within-subject factors. Both participants (*F*_1_) and items (*F*_2_) were treated as random factors in the analyses.

There was a significant main effect of task in the RT analyses, *F*_1_(1, 31) = 369.27, *p* < .001, η_p_^2^ = .923; *F*_2_(1, 23) = 440.02, *p* < .001, η_p_^2^ = .950. This stemmed from the faster RTs in the font size judgment than in the referred-object judgment. The main effect of congruency was not significant, *F*_1_(1, 31) = 0.224, *p* = .639, η_p_^2^ = .007; *F*_2_(1, 23) = 0.120, *p* = .732, η_p_^2^ = .005). The interaction between task and congruency was not significant, *F*_1_(1, 31) = 0.002, *p* = .962, η_p_^2^ = .001; *F*_2_(1, 23) = 0.197, *p* = .662, η_p_^2^ = .008.

**Figure 7.**
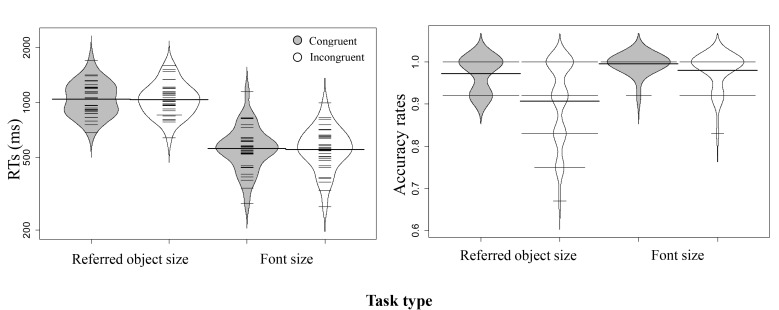
Distribution of participants’ reaction times (RTs) (ms) and accuracy rates (ARs) in Experiment 3a (conventions as in Figure 2). RT: Refer-Congruent; *M* = 1064.85, *SE* = 40.53. Refer-Incongruent; *M* = 1057.17, *SE* = 39.48. Font-Congruent; *M* = 580.08, *SE* = 29.96. Font-Incongruent; *M* = 573.61, *SE* = 27.29. AR: Refer-Congruent; *M* = 0.971, *SE* = 0.007. Refer-Incongruent; *M* = 0.906, *SE* = 0.017. Font-Congruent; *M* = 0.995, *SE* = 0.004. Font-Incongruent; *M* = 0.979, *SE* = 0.007.

In the AR analyses, there was a significant main effect of task, *F*_1_(1, 31) = 28.694, *p* < .001, η_p_^2^ = .481; *F*_2_(1, 23) = 20.690, *p* < .001, η_p_^2^ = .478. This stemmed from the higher AR in the font size judgment than in the referred-object judgment. A significant main effect of congruency was obtained, *F*_1_(1, 31) = 15.787, *p* < .001, η_p_^2^ = .337; *F*_2_(1, 23) = 21.061, *p* < .001, η_p_^2^ = .478. Importantly, the interaction between task and congruency was significant, *F*_1_(1, 31) = 6.059, *p* = .020, η_p_^2^ = .163; *F*_2_(1, 23) = 8.342, *p* = .008, η_p_^2^ = .266. We further conducted planned simple effect analyses and found that participants’ accuracy was significantly higher in the congruent than in the incongruent condition for the object task, *F*_1_(1, 31) = 12.24, *p* = .001, η_p_^2^ = .875; *F*_2_(1, 23) = 18.13, *p* < .001, η_p_^2^ = .454, and participants’ accuracy was marginally higher in the congruent than in the incongruent condition for the font size task, *F*_1_(1, 31) = 3.930, *p* = .056, η_p_^2^ = .117; *F*_2_(1, 23) = 3.800, *p* = .063, η_p_^2^ = .130 (see Table 4, Figure 7). Furthermore, we performed paired-sample *t*-tests to test the congruency effect: The difference between congruent and incongruent condition. The results showed that the congruency effect was more significant, *t*(31) = 2.462, *p* = .020, *d* = 0.620, in the referred-object size task (*M* = 0.065) than in the font size task (*M* = 0.015).

As we found in AR analyses, there were size-congruency effects in the referred-object task, which were the same as in Experiment 1a. There were also size-congruency effects in the font size task, which were different from the results of Experiments 1b and 2. We assumed that whether semantic processing affects perceptual processing might be modulated by other factors, such as the degree of semantic activation. We found that the degree of semantic activation was different among Experiments 1b, 2, and 3a. In Experiment 1b, participants might have ignored the semantics and processed the word font only so that the semantics were not affected by perceptual processing. When the level of semantic activation was enhanced, in the mixed task in Experiment 3a, the size-congruency effect was found in the AR analyses. In order to address whether semantic activation modulated the bidirectional link between perceptual and conceptual processing, Experiment 3b utilized a similar paradigm but presented the task cue after the paired words.

## Experiment 3b

### Method

#### Participants

Another 32 university students (*M*_age_ = 21 years, *SD* = 1.38, 19 were female) from South China Normal University, Guangzhou, China, participated in this experiment. Other aspects were the same as those in Experiment 3a.

#### Materials and Procedure

**Figure 8.**
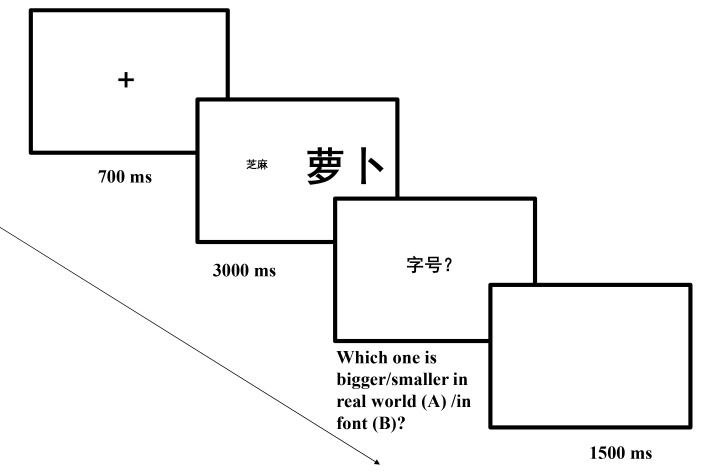
Sequence of events in a trial of Experiment 3b (萝 卜 means *radish*, 芝 麻 means *sesame seed* and 字 号 means font?).

Materials were identical to those of Experiment 1a. The design was the same as in Experiment 3a, but unlike Experiment 3a, the displayed cue appeared after the pair of words. Each trial began with a red cross that was presented for 700 ms at the center of the screen. Afterwards, a pair of words appeared at the center of the screen for 3 s. One word was presented in a larger font size; the other, in a smaller font size. Meanwhile, one word referred to a larger object in reality while the other referred to a smaller object. Then, a task cue (i.e., “concept?” or “font?”) was presented at the center of the screen. When participants saw “concept?”, they judged which word referred to the larger or smaller object in reality. When participants saw “font?”, they judged which word was presented in larger or smaller font size. This task cue was presented for 5 s or until the participants responded. Half of the participants first judged which one was smaller, and the other half first judged which one was bigger. Participants pressed the *c* or *m* key on the keyboard to judge which word referred to a larger/smaller object in reality or which word was presented in larger/smaller font size. After a 1,500 ms blank screen, the next trial was released (see Table 5, Figure 8).

#### Design and statistical analyses

The study employed a 2 (task: referred-object judgment task vs. font size judgment task) × 2 (consistency: congruent vs. incongruent) within-subject design. The statistical analyses were the same as those used for Experiment 1a.

### Results

Data from five participants were removed because of low accuracy (< 80%). To improve the stability and the validity, we collected data from another five participants in order to have 32 valid participants’ data. RT data with erroneous trials were deleted (4.42%), and RTs beyond two SD were excluded from the analyses (4.55%). All AR data were included for further analysis.

Trimmed RTs and ARs were submitted to two separate two-way repeated measures ANOVAs with task (referred-object size judgment vs. font size judgment) and congruency (congruent vs. incongruent) as within-subject factors. Both participants (*F*_1_) and items (*F*_2_) were treated as random factors in the analyses.

There was a significant main effect of task in the RT analyses, *F*_1_(1, 31) = 4.91, *p* = .034, η_p_^2^ = .13; *F*_2_(1, 23) = 15.04, *p* = .001, η_p_^2^ = .39. This effect stemmed from the slightly faster RTs for font size judgments than for referred-object judgments. A significant main effect of congruency was obtained, *F*_1_(1, 31) = 25.02, *p* < .001, η_p_^2^ = .45; *F*_2_(1, 23) = 46.29, *p* < .001, η_p_^2^ = .68. This indicated that participants’ responses were faster in the congruent than in the incongruent condition. The interaction between task and congruency was not significant, *F*_1_(1, 31) = 0.11, *p* = .746, η_p_^2^ = .003; *F*_2_(1, 23) = 1.56, *p* = .223, η_p_^2^ = .064. To address our research questions, we further conducted planned, simple effect analyses. We found that participants’ responses were faster in the congruent than in the incongruent condition regardless of task, for the referred-object task, *F*_1_(1, 31) = 7.35, *p* = .011, η_p_^2^ = .191; *F*_2_(1, 23) = 13.33, *p* = .001, η_p_^2^ = .36, and for the font size task, *F*_1_(1, 31) = 39.68, *p* < .001, η_p_^2^ = .561; *F*_2_(1, 23) = 64.44, *p* < .001, η_p_^2^ = .73. Furthermore, we performed paired-sample *t*-tests to test the congruency effect: the difference between congruent and incongruent conditions. The result showed that there was no significant effect difference, *t*(31) = .327, *p* = .746, *d* = 0.010, between referred-object size judgment (*M* = 154.54) and font size judgment (*M* = 174.45).

**Figure 9.**
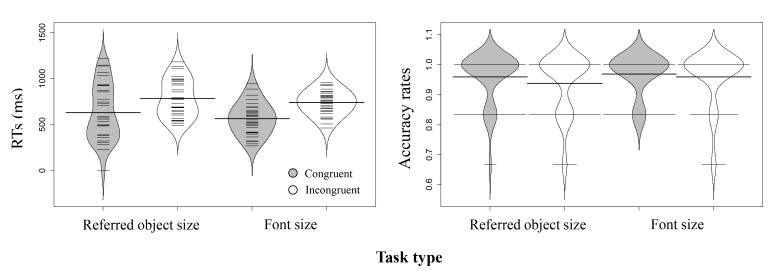
Distribution of participants’ reaction times (RTs) (ms) and accuracy rates (ARs) in Experiment 3b (conventions as in Figure 2). RT: Refer-Congruent; *M* = 627.55, *SE* = 39.05. Refer-Incongruent; *M* = 782.09, *SE* = 36.42. Font-Congruent; *M* = 564.12, *SE* = 30.94. Font-Incongruent; M = 738.58, *SE* = 22.56. AR: Refer-Congruent; *M* = 0.958, *SE* = 0.015. Refer-Incongruent; *M* = 0.937, *SE* = 0.018. Font-Congruent; *M* = 0.969, *SE* = 0.012. Font-Incongruent; *M* = 0.958, *SE* = 0.017.

In the AR analyses, the main effect of task was not significant, *F*_1_(1, 31) = 0.74, *p* = .395, η_p_^2^ = .002; *F*_2_(1, 23) = 1.302, *p* = .266, η_p_^2^ = .05. The main effect of congruency was not significant, *F*_1_(1, 31) = 1.13, *p* = .296, η_p_^2^ = .03; *F*_2_(1, 23) = 0.896, *p* = .354, η_p_^2^ = .03. The interaction between task and congruency was also not significant, *F*_1_(1, 31) = 0.139, *p* = .712, η_p_^2^ = .004; *F*_2_(1, 23) = 0.13, *p* = .714, η_p_^2^ = .006 (see Figure 9). These results confirmed that there was no speed-accuracy trade-off effect.

### Comparison of Experiments 3a and 3b

In order to test our prediction that the degree of semantic activation played an important role in the relationship between semantic and perceptual processing, we compared data from Experiments 3a and 3b in an ANOVA. Experiment (Experiment 3a vs. Experiment 3b) was included as a between-subjects factor. Trimmed RTs and ARs were submitted to three-way mixed ANOVAs, with task (referred object size judgment vs. font size judgment) and congruency (congruent vs. incongruent) as within-subject factors. Both participants (*F*_1_) and items (*F*_2_) were treated as random factors in the analyses.

There was a significant main effect of experiment in the RT analyses, *F*_1_(1, 62) = 11.22, *p* = .001, η_p_^2^ = .15; *F*_2_(1, 46) = 113.77, *p* < .001, η_p_^2^ = .71. This effect stemmed from faster RTs in Experiment 3b than in Experiment 3a. The interaction between experiment and congruency was significant, *F*_1_(1, 62) = 22.56, *p* < .001, η_p_^2^ = .267; *F*_2_(1, 46) = 34.92, *p* < .001, η_p_^2^ = .43. We further conducted planned, simple effect analyses and found that participants’ responses were faster in the congruent than in the incongruent condition in Experiment 3b, *F*_1_(1, 62) = 41.48, *p* < .001, η_p_^2^ = .42; *F*_2_(1, 46) = 65.48, *p* < .001, η_p_^2^ = .98. However, the effect of congruency on speed of participants’ responses was not significant in Experiment 3a, *F*_1_(1, 62) = 0.08, *p* = .783, η_p_^2^ = .001; *F*_2_(1, 46) = 0.07, *p* = .79, η_p_^2^ = .001.

In the AR analyses, the main effect of experiment was not significant, *F*_1_(1, 62) = 0.659, *p* = .420, η_p_^2^ = .01; *F*_2_(1, 46) = 0.923, *p* = .342, η_p_^2^ = .02. The interaction between experiment and congruency was not significant, *F*_1_(1, 62) = 1.96, *p* = .167, η_p_^2^ = .03; *F*_2_(1, 46) = 1.731, *p* = .195, η_p_^2^ = .03.

### Discussion

In Experiments 3a and 3b, we found that conceptual processing was influenced by perceptual processing. Specifically, in the referred-object size task, participants were not required to process font size, which was an irrelevant dimension in the task. We found that font size significantly affected conceptual processing, which might suggest that perceptual processing affects semantic processing. When concepts that referred to larger objects in reality were presented in larger font sizes, participants’ responses for these concepts were faster (in Experiment 3b) or more accurate (in Experiment 3a) than when presented in smaller font sizes. When concepts that referred to smaller objects in reality were presented in smaller font sizes, participants’ responses were faster and more accurate than when presented in larger font sizes.

In addition, we found that perceptual processing was affected by conceptual processing, which depends on the degree of semantic activation. By adopting a mixed task in Experiment 3a and a dual task in Experiment 3b, we found a size congruent effect in the font size task in the AR analysis in Experiment 3a, in which participants were asked to execute the task signaled by the preceding cue. When the degree of semantic activation was enhanced by presenting a delayed task cue in Experiment 3b, the size-congruency effect appeared. These results suggested that whether semantic processing affects perceptual processing and vice versa might be modulated by the degree of semantic activation.

## General discussion

The present study tested the interaction between objects as referents and their symbols (words) and further investigated dynamic variations of this interaction under the degree of semantic activation. Experiment 1a confirmed that perceptual processing affected conceptual processing. When words referring to larger (or smaller) objects were presented in larger (or smaller) font sizes, participants’ conceptual judgments were more accurate. This effect is known as the size-congruency effect. In Experiment 1b, the same Stroop-like paradigm was used except for adopting a font judgment task. We did not find such size-congruency effect. Considering the font task was relatively easy and word meanings might not have been activated, we added a recognition task in Experiment 2. This forced participants to retrieve word meanings. However, once again, the results did not show the size-congruency effect. Therefore, in Experiment 3a, a mixed task was used to further activate semantic processing. As a result, we found that perceptual processing affected conceptual processing, and vice versa. To further test the impact of semantic activation, we explored a dual task and presented the task cue after the presentation of paired words. Consequently, participants had to process word meanings because they did not know which task (i.e., semantic or font) they were expected to do next. The results also showed that perceptual processing affected conceptual processing, and vice versa. Thus, the above mentioned results indicated that the bidirectional relationship between conceptual and perceptual processing might depend on the degree of semantic activation. Conceptual and perceptual processing might interact in certain situations.

 Experiments 1a and 3 indicated that conceptual processing was significantly affected by perceptual processing that supports PST. The current results were similar to previous findings. For example, Tang, Ye, and Du (2015) employed a Stroop paradigm to investigate the metaphoric congruency effect between font size and power valence. They selected powerless and powerful words and presented them in small or large font sizes. Participants judged which word was more (or less) powerful than the other. They found that the participants’ responses were faster when powerful (or powerless) words were presented in large (or small) font size. In another study, Henik and Tzelgov (1982) asked participants to decide which digit (5 vs. 3) in each pair was larger in a numerical or in a font size judgment task. Related to the current results, RTs were faster in the congruent condition and slower in the incongruent condition. 

The current study also suggested that conceptual processing influenced perceptual processing, and the relationship between them was modulated by semantic activation. In Experiment 1b, the font size task was too easy and participants ignored the semantic meaning of the words. This may also be true in Experiment 2. The results of these two experiments were similar to the results of Paivio’s (1975) study in which participants responded fastest for congruent and slowest for incongruent picture pairs, while they responded similarly for congruent and incongruent word pairs. Hence, semantic processing may be necessary for a size-congruency effect. We tested this hypothesis in Experiment 3.

 However, when the degree of semantic activation was increased in Experiment 3, the effect was found, suggesting that the degree of semantic activation was vital for the relationship between conceptual and perceptual processing. Semantic activation implicated the re-enactment of sensorimotor information, which made an effect on perceptual processing. Embodied semantics means that concepts are represented in the brain within the same sensory-motor circuitry on which the enactment of that concept relies (Aziz-Zadeh & Damasio, 2008). Previous research has shown that the relationship is modulated by task and semantic activation (Connell & Lynott, 2013; D’Arcais et al., 1985; Siakaluk et al., 2008). For example, Huang and Tse (2015) found that conceptual processing only affected spatial processing when the spatial task was simultaneously performed with a 4-dot-position visuospatial rehearsal task, in which participants remembered the four successive dot positions. This finding may indicate that the effect of conceptual on perceptual processing is modulated by concomitant tasks. In Experiment 2, participants only judged font sizes of word pairs. On the contrary, in Experiment 3, participants simultaneously judged font sizes and in-reality sizes of referred objects. Thus, the task in Experiment 3 needed more semantic processing than that in Experiment 2, which resulted in higher-level semantic activation. As a result, we only found a size-congruency effect in Experiment 3. Grounded cognition proposes that sensorimotor information underlies conceptual processing. In the current study, the semantic information activated automatically in the reference object task (conceptual processing), but not in the font size task (perceptual processing). When a word was processed during a conceptual task, it first activated word formation, and then a situated simulation to represent its meaning. Moreover, the situated simulation to represent its meaning would have only started if semantic activation was strong enough (Barsalou, 1999; Glenberg & Kaschak, 2002). Therefore, the current study suggested that the effects of conceptual on perceptual processing were modulated by semantic activation. 

Moreover, we wanted to emphasize the interaction that we observed between task and congruency in Experiment 3a. Here, participants’ accuracy was higher in the congruent than in the incongruent condition regardless of tasks. In contrast, the results showed a significant effect of congruency in the referred-object task, but a marginal effect of congruency in the object size task. According to the PST, conceptual processing is based on perceptual symbols. Perceptual processing is the foundation of cognitive processing and supports other higher level cognitive processing (e.g., conceptual processing). This may be the reason that we found a difference between the two tasks. Furthermore, the *t*-tests showed that the congruency effect of the referred-object task was significantly stronger than that of the font size task, while there was no such effect in Experiment 3b. This indicated that processing goal (known vs. unknown in Experiments 3a and 3b) appeared to have a special role in the interaction between an object as a referent and its symbol. In Experiment 3a, the task cue appeared before the targets; thus participants could prepare to respond to only one task. In Experiment 3b, the task cue appeared after the targets; thus participants had to prepare for these two tasks simultaneously. Maybe this interaction in Experiment 3a is due to shared response codes. For instance, it is possible that participants also implicitly compared the font sizes when they were required to compare conceptual sizes; and because the two types of sizes used the same response codes, there was a conflict for the incongruent trials, resulting in the observed size-congruency effect.

 In contrast, PST suggests that the size-congruency effect arises because conceptual and perceptual representations share the same processing system (Goldberg, Perfetti, & Schneider, 2006; Simmons et al., 2007). Perceptual and conceptual information would interact with each other. When people process concepts, the related perceptual information is activated. We conducted the current study in an attempt to clarify the relationship between size’s conceptual and perceptual processing. According to PST, the internal representation of concrete concepts is based on perceptual symbols. This might be the reason why in the current study perceptual processing automatically affected concepts, whereas concept processing modulated by the semantic activation affected perceptual processing ( Yeh & Barsalou, 2006). Perceptual processing is fundamental in cognitive processing and supports other higher level cognitive processing (e.g., conceptual processing). Therefore, perceptual processing automatically affects conceptual processing, whereas conceptual processing affects perceptual processing only in special contexts. Finally, the present study is in line with previous studies on other domains. Some studies have shown an interaction between conceptual and perceptual processing, such as digit size in Arabic numerals (Besner & Coltheart, 1979; Girelli, Lucangeli, & Butterworth, 2000; Pansky & Algom, 1999; Santiago & Lakens, 2015) or animal size where the stimuli were pairs of animal names (Rubinsten & Henik, 2002). 

 The current study also contributed to the existing evidence of opposite findings in this area of research (see Paivio, 1975, vs. Rubinsten & Henik, 2002). We assume that task related factors might influence the size-congruency effect. In our study, with the degree of semantic activation increasing, the congruency effect was found, suggesting that the degree of semantic activation was vital for the relationship between conceptual and perceptual processing. Previous research has shown that the relationship was modulated by task and semantic activation (Connell & Lynott, 2013; Siakaluk et al., 2008). Alternatively, task difficulty might be another experimental factor that could explain why our findings were similar to the study of Rubinsten and Henik (2002) but not to the study of Paivio (1975) . Much research has provided evidence that task difficulty plays an important role in cognitive tasks ( Huber, 1985; Kaspar & Vennekötter, 2015; Robinson, 2001). The interleaved tasks in Experiment 3 were harder than the other tasks, including the recognition task in Experiment 2 and the single task in Experiment 1b. This may be a reason why we found a size-congruency effect in Experiment 3 but not in Experiments 1b and 2. 

 Finally, we also assumed the different findings were due to the task goal. Other studies have reported that task goals were related to semantic priming, thus influencing task performance. For example, Hoedemaker and Gordon (2014) used a priming paradigm and found that the activation of numerical magnitude representations was encoding-based as well as goal-driven, while the activation of size information associated with words was goal-driven and did not occur automatically during encoding. These findings are consistent with our current results that task-related factors might affect the relationship between conceptual and perceptual processing. Exploring the linguistic and embodied nature of conceptual processing, Louwerse and Jeuniaux (2010) reported that the task modified the effect of embodied and linguistic factors in relation to the response of picture and word judgment. The current studies used different tasks (i.e., a recognition task in Experiment 2, an anticipated task in Experiment 3a, and an uncertain task in Experiment 3b) to explore the relationship between conceptual and perceptual processing. The results further showed that task related factors might have also influenced participants’ performance. 

 Our study is in line with others and provides new evidence for grounded cognition. Nevertheless, several questions remain. First, the nature of the relationship between conceptual and perceptual processing should be investigated in other domains, such as color and shape. Second, follow-up studies should test the time course of the size-congruency effect. Finally, it might be valuable to investigate the relationship between perceptual and conceptual processing, not only in concrete but also in abstract concepts. The current material was composed of concrete concepts; and it is likely that participants had substantial experiences with concrete concepts. As to abstract concepts, there is evidence that sensory motor information is activated when processing affective concepts (Barsalou, 1999, 2008, 2012) and moral concepts (Hill & Lapsley, 2009; Meier, Sellbom, & Wygant, 2007; Williams & Bargh, 2008). 

In summary, the current findings enriched grounded cognition theory, especially the PST. A strong hypothesis of grounded cognition holds that perceptual and conceptual processing are essentially of the same kind, predicting a bidirectional relationship between conceptual processing and perceptual processing regardless of the task relevance of semantic information. However, our results indicate that the relationship between conceptual and perceptual processing might be bidirectional and might be further modulated by semantic activation. Therefore, the current findings provide new evidence for grounded cognition.

## Footnotes

1
We have examined the variable of target type (bigger task vs. smaller task) in all three experiments, and the results showed that this variable did not influence our results.

## Appendix A

Twenty-four pairs of words were used in all 3 experiments.
